# A hybrid physics-informed neural and explainable AI approach for scalable and interpretable AQI predictions

**DOI:** 10.1016/j.mex.2025.103597

**Published:** 2025-08-29

**Authors:** Sai Varun Chandrashekar, Firoz Khan, Sunaina Sridhar, Rajesh Kumar Dhanaraj, Balamurugan Balusamy, Dragan Pamucar, Malathy Sathyamoorthy

**Affiliations:** a Department of Computer Science and Engineering, Sathyabama Institute of Science and Technology, Chennai, India; b Assistant Professor in Centre for Information and Communication Sciences, Ball State University, USA; c Delivery Executive, Google LLC, Chicago, USA; d Symbiosis Institute of Computer Studies and Research (SICSR), Symbiosis International (Deemed University), Pune, India; e School of Engineering and IT, Manipal Academy of Higher Education, Dubai Campus, Dubai, UAE; f Széchenyi István University, Győr, Hungary; g Department of Information Technology, KPR Institute of Engineering and Technology, Coimbatore, India

**Keywords:** Air quality index, Air pollution classification, Environmental monitoring, Explainable ai, Interpretable machine learning, Physics-informed neural networks

## Abstract

*Air Pollution is a critical environmental issue affecting public health, climate, and ecosystems. However, accurately predicting and classifying Air Quality Index (AQI) levels across different regions remains a challenging task due to the complex nature of air pollution patterns. Conventional and ensemble ML and DL models often fail to capture the physical laws goverming the air pollution, which leads to inaccurate predictions. This study addresses these issues by introducing an approach that employs Physics-Informed Neural Networks (PINN) with Explainable AI (XAI) techniques for AQI classification (AirSense-X). The proposed approach utilizes PINN for regression, along with mapping for classification and XAI for interpretation. PINN ensures that the model learns from physical laws governing air quality rather than relying solely on data. The dataset utilized in this study is a publicly available dataset containing the AQI data at daily levels from various stations across multiple cities in India. The proposed AirSense-X approach achieves an accuracy of 98 %, with 97 % precision, 95 % recall, and an F1 score of 0.96, ensuring reliability. Similarly, the confusion matrix for the proposed approach indicated that the model correctly classified 21,306 and misclassified 268 instances.*

*The key focuses of this study include:*

• *Introducing a novel approach, AirSense-X, which employs PINN for accurate AQI prediction and XAI for enhanced interpretability. Additionally, the study also involves comparative analysis with conventional and ensemble ML and DL models.*
• *Employing structure mapping technique for classification based on the predicted AQI values.*
• *Integrating physical laws governing air pollution using a PINN model enhances prediction accuracy and ensures that the model learns beyond relying on data-driven insights.*

## Graphical abstract

**Figure:**
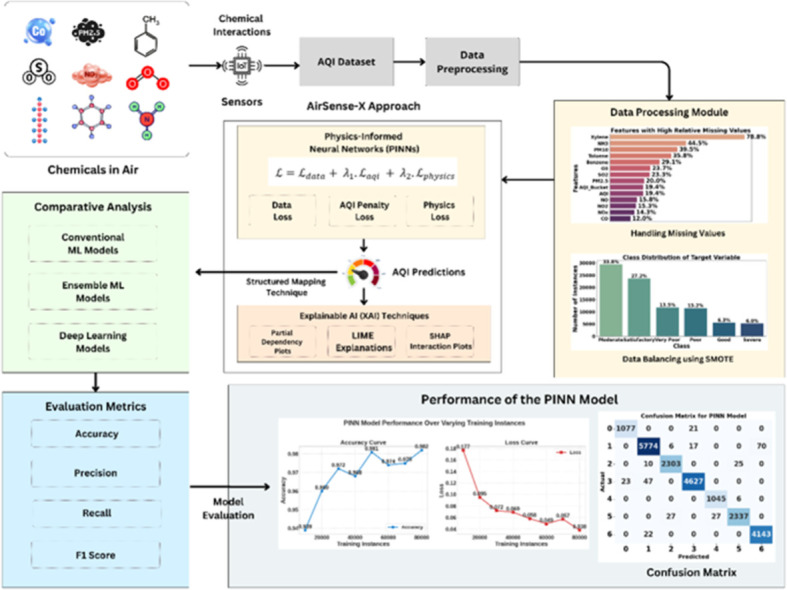

**Specifications table**

**Table:** 

**Subject area**	Computer Science
**More specific subject area**	AI in Sustainability
**Name of your method**	AirSense-X
**Resource availability**	https://www.kaggle.com/datasets/rohanrao/air-quality-data-in-india

**Background**

## Introduction

*Air Pollution has become one of the most significant environmental challenges affecting global health, climate, and ecosystems. According to the World Health Organization (WHO), air pollution is responsible for millions of premature deaths, primarily due to respiratory and cardiovascular diseases [* *1* *,* *2* *]. The AQI is a numerical scale that ranges from 0 to 500, commonly used to measure and communicate the quality of air, with levels indicating whether the air is considered healthy or hazardous [* *3* *]. However, accurately predicting AQI levels across different geographical regions is a complex task. These multiple factorial influences make it challenging to develop a precise model for AQI prediction [* *4* *]. The existing systems for AQI prediction face significant challenges in capturing complex interactions and physical laws that govern air pollution patterns [* *5* *]. Conventional and ensemble machine learning (ML) models are utilized, but they often fail to incorporate the physical laws governing air pollution [* [6], [7], [8] *]. These limitations lead to inaccurate predictions, particularly in regions with varying geographical features and pollution sources. This highlights the critical need for an approach that can integrate both data-driven and physical law-based constraints to improve AQI predictions.*

*The study introduces a novel approach for addressing the issues with existing solutions, combining AirSense-X with PINN for regression and XAI techniques to enhance reliability and model interpretability. This approach utilizes physical laws to enhance AQI classification. The PINN model ensures that insights into the physical relationships between air pollutants and AQI are extracted, thereby avoiding dependence on data alone. Meanwhile, a structured mapping technique is used for classification, analyzing the patterns of pollution concentration in the data. Finally, XAI techniques are integrated to enhance the transparency of the model, providing explanations about how specific pollution variables influence AQI prediction.*

*The key focuses of this study include:*

• *Introducing a novel approach, AirSense-X which employs PINN for accurate AQI prediction.*
• *Employing structure mapping technique for classification, based on the predicted AQI values.*
• *Integrating physical laws governing air pollution using PINN model which enhances the prediction accuracy and ensures that the model learns beyond relying on data-driven insights.*
• *Improving model interpretability using XAI techniques to provide model transparency.*
• *Additionally, comparing the performance of the proposed AirSense-X approach with conventional and ensemble ML and DL models.*

### Related works

The degradation of air quality poses a severe threat to public health, particularly in densely populated regions, where rapid urbanization and industrial activities contribute significantly to increases levels of pollutants such as PM2.5, PM10 and CO. Traditional methods for air quality prediction struggle with the highly dynamic and spatiotemporal nature of pollution, necessitating the requirement of ML and DL models for enhanced prediction accuracy. Several studies have employed ML and DL models for enhanced prediction accuracy. AQI has increasingly benefited from the application of ML and DL models due to their ability to analyze complex patterns and generate accurate predictions. The comprehensive study conducted in Zabol employed several ML and DL models, including random forest, K-Nearest Neighbor (KNN), Weighted KNN, SVM, ANN, LSTM, RNN and CNN for showing CNN’s superios performance with a higher accuracy for AQI classification. However, the study was limited by inconsistent and incomplete environmental data, ensuring that the model is less strong in real-time settings [9]. In a study on Indian cities like New Delhi and Kolkata, models like Random Forest and CatBoost models showed enhanced performance with SMOTE balancing but the absence of meteorological features and dependence on synthetic data limited the model’s generalization ability [10]. A comparative study between SVM and KNN model showed improved performance with hybrid datasets, but the lack of diversity in model selection and consideration of only limited pollutants, reduced adaptability [11]. Another study using classical ML models for forecasting AQI in Azamgarh showed the Feedforward Neural Network (FNN) performin nest but failed to include meteorological factors and suffered from overfitting due to small dataset size [12]. A study using Grey Wolf Optimization with Decision Tree (GWO-DT) achieved high accuracy but the lac of temporal feature handling and static feature dependence reduced effectiveness in dynamic environmental scenarios [13]. Another study using classical ML models for predicting AQI in The authors of [14] proposed ensemble boosted tree (EBOT) and ensemble bagged tree (EBAT) in Makkah, where EBOT achieved the highest accuracy of 97.4 %. However, a key limitation ML-based approaches is their reliance on handcrafted features, which may not always capture complex pollutant interactions and long-term dependencies effectively. ML models for AQI prediction are often limited by feature engineering dependency, difficulty in capturing time-series patterns and the lack of adaptability to unseen data. They also struggle to handle spatiotemporal variability in air pollution datasets which limits their scalability.

Similarly, DL architecture such as CNN and LSTM have shown superior performance in capturing intricate patterns within air pollution data. DL models are offered to offer better handling of sequential and non-lineat patterns. The study involves comparative analysis of LSTM, GRU, Bi-LSTM and CNN for hourly AQI prediction but it performed poorly for daily AQI prediction and lacked incorporation of external influencing factors like weather or traffic [15]. Another study from China employed five DL models to capture AQI prediction but the complex architecture led to high computational cost and difficulty in model interpretability, making real-time deployment challenging [16]. One of the study have implemented a DL named AQE-Net for air quality prediction. Despite these advancements, DL models often suffer from high computational costs and the need for extensive labeled datasets. Furthermore, most ML and DL models rely purely on data-driven approaches, often neglecting the physics-based laws that govern air pollution, which can lead to overfitting. Despite their power, DL models face general limitations such as the need for large labeled datasets, high chances of overfitting, black-box nature of model decisions and high computational cost which constraint their practical utility in resource-limited regions.

Hybrid models that integrate ML and DL techniques have been explored to overcome these limitations. This study [[17], [18]] proposed a hybrid approach integrating LSTM and GRU models to predict AQI for PM2.5. Hybrid models aim to overcome individual weaknesses by combining techniques. One of the study proposed a hybrid ANN-LSTM model over 60,000 samples of pollutants and achieved an accuracy of 94.87 % across metrics like MSE and R2. While the performance outperformed traditional models like Linear Regression, Random forest and Decision Trees, the system’s complexity and dependency on large-scale, high-quality data for training made it less practical for under-monitored regions [19]. Another extensive review compared conventional models like ARIMA and ML methods with hybrid approaches such as GA-ELM and LSTM-CNN. A further study used hybrid techniques combining Input Variable Selection (IVS), regression and ML for predicting PM1, PM2.5 and PM10 concentrations using a two-year dataset from Romania. Although R^2^ socre values exceeded 0.93, the hybrid mdel’s multistage structure, high dimensionality and variance in prediction precision introduced complexity in deployment and scalability [20]. While the hybrid models improved accuracy while employing the strengths of various techniques as they are computationally inensive, difficult to fine-tune and less interpretable which can hinder the real-time implementation in public health settings.

Table 1 summarises the key existing studies on AQI prediction using conventional ML, DL and hybrid approaches using data collected from different cities. The GWO algorithm was employed with Decision Trees, achieving enhanced accuracy but depending on conventional ML models without integrating physical laws or explainability [13]. Similarly, a hybrid approach, combining LSTM with GRU, was trained using Delhi's AQI data, showing enhanced predictive performance but lacking interpretability and inclusion of domain knowledge [18]. The study focuses on improving predictive accuracy using a hybrid approach, combining ARIMA and Random Forest models and XAI techniques for enhanced interpretability. Still, it lacks the integration of physical laws, which limits its reliability [21]. This study focuses on maintaining the trade-off between higher accuracy, enhanced explicability and reduced computational overhead. Additionally, integrating physical laws using PINN enhanced the model's reliability, accuracy, and strength. Overall, the AirSense-X approach addresses existing works' interpretability and reliability gaps, making it a more insightful tool for air quality assessment.

**Table 1:** Comparative analysis of state-of-the-art models with AirSense approach.

Reference	Dataset	Methodology	Results	Research Gap
[13]	It contains hourly and daily AQI data from major Indian cities (2015–2020).	Grey Wolf Optimizaion (GWO) is combined with Decision Tree algorithm for enhanced prediction of AQI.	The proposed approach achieved an accuracy range of 88.98 to 97.68, for different cities in India.	The study depends on conventional ML models and optimization techniques, neglecting physical laws or explicability, limiting model’s interpretability.
[18]	It contains the AQI data, specifically for Delhi.	The proposed hybrid approach includes LSTM and Gated Recurrent Unit (GRU) to predict the AQI data.	The hybrid approach shows superior performance with the MAE value of 36.11 and R^2^ score value of 0.84.	The study focuses on enhancing the performance but lacks interpretability and integration of physical laws.
[21]	It is sourced from Kaggle, containing daily and hourly air quality, collected from different Indian cities and monitoring cities.	The hybrid approach includes Random Forest Regression and Autoregressive Integrated Moving Average (ARIMA) to enhance AQI prediction accuracy along with XAI techniques.	The hybrid approach achieves MSE of 508.46 and R^2^ score of 0.94.	The study focuses of enhancing accuracy along with explainability but fails to integrate physical laws for enhanced reliability.

### Research gaps

RQ1: How can physical laws governing air pollution dynamics be incorporated into AQI prediction models to enhanced generalization ability and accuracy?

Existing works depend heavily on data-driven approaches and handcrafted features, neglecting physics-based constraints. This study addresses this issue by integrating PINN which enables hybrid learning approaches that combine data-driven approaches and physical-laws based approaches.

RQ2: How can XAI techniques be effectively integrated into AQI prediction models to enhance interpretability without sacrificing performance?

Most of the existing studies employ the use of black box models for AQI prediction. This study address this gap by employing XAI techniques, offering model interpretability that enhance trust and transparency in the model’s predictions.

RQ3: What is the impact of integrating physics-informed constraints with DL in reducing overfitting and enhancing performance in under-monitored regions?

Existing works often require large, high-quality datasets and suffer from overfitting. This paper avoids this situation by combining physics-based modeling with learning, enabling generalization even in limited data.

RQ4: How can AQI models be designed to handle both classification and regression tasks effectively within a unified architecture?

Previous works usually focus on either classification or regression. The proposed approach in this study involves the use of PINN for regression along with structured mapping for classification for multi-objective learning.

RQ5: To what extent can incorporating meterological and external factors along with pollutant data improve the spatiotemporal accuracy of AQI predictions?

Most of the cited studies neglect physical factors influencing the variable. The proposed methodology in this study ensures comprehensive feature inclusion, enhancing predictive capability in dynamic environments.

This study introduces a novel approach to addressing all the issues with the existing solutions, AirSense-X combined PINN for regression and CNN for classification along with XAI techniques for model interpretability. The hybrid model ensures the usage of physical laws to improve AQI classification.

## Method details

### Dataset description

This study utilizes a publicly available dataset from Kaggle, containing AQI and air quality data collected daily from various stations across multiple cities in India [22]. The dataset includes five categories: Poor, Very Poor, Satisfactory, Good, and Severe. It contains approximately 1,10,000 instances and 14 attributes, including the target variable. The dataset is divided into an 80 % training set and a 20 % testing set.

**Fig. 1:**
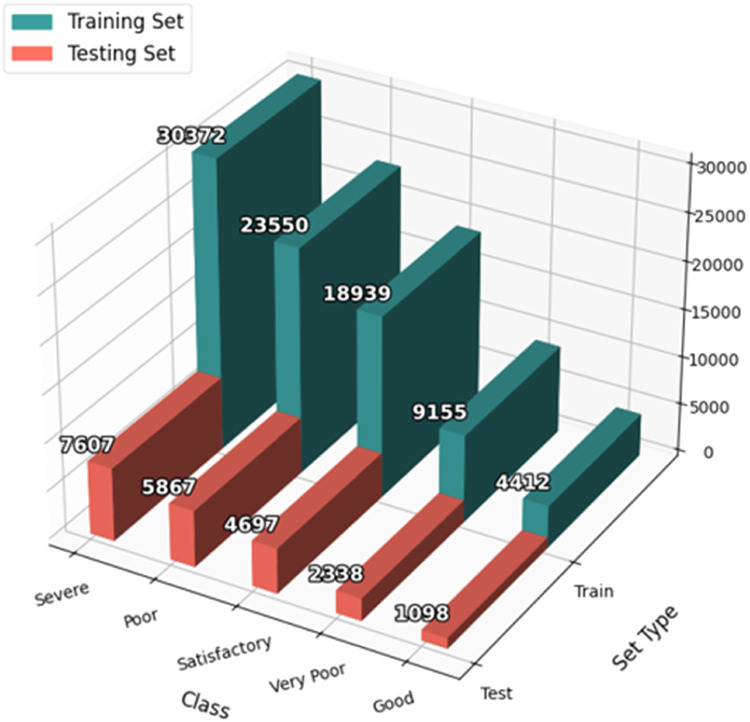
Distribution of AQI categories in training and testing sets.

Table 2 and Fig. 1 show the distribution of AQI categories in both the training and testing sets. For each AQI class, the number of instances in the training and testing set is provided. The training set has a larger number of instances for all classes, as it comprises 80 % of the total data, while the testing set contains the remaining 20 % of the data.

**Table 2:** Dataset summary.

Class	Training Set	Testing Set
Severe	30,372	7607
Poor	23,550	5867
Satisfactory	18,939	4697
Very Poor	9155	2338
Good	4412	1098

The study employs a publicly available secondary dataset sourced from Kaggle. It contains nearly 8785 instances, recorded throughout 2024 across three globally distributed cities [23]. The dataset captures key pollutants such as CO, CO2, NO2, SO2, O3, PM2.5 and PM10, along with the AQI. The geographical diversity of the dataset makes this dataset ideal for testing the AirSense-X approach’s generalisation ability. This dual dataset strategy would allow the AirSense approach to adapt effectively to varied urban environments and maintain predictive accuracy across different conditions.

### System architecture

**Fig. 2:**
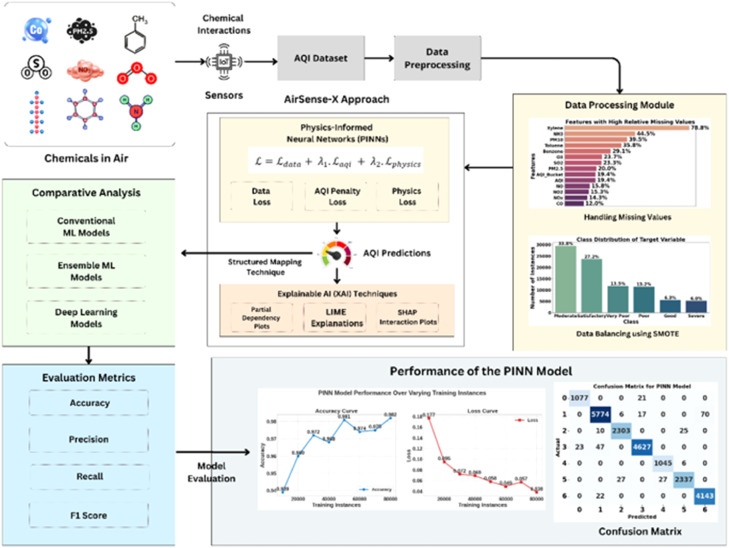
System architecture of AirSense-X approach.

The system architecture section outlines the proposed approach, and Fig. 2 provides a visual diagram for a clearer understanding of the system’s architecture. The AirSense-X is an approach developed for AQI prediction and analysis that combines a PINN model with XAI methods. Initially, data is collected from sensors that detect various chemicals, including PM2.5, CO, and others. These sensor readings are present in the AQI dataset that is collected from various stations in India. The data then undergoes data preprocessing, including handling missing values and addressing class imbalance using SMOTE. The primary component of this approach is the PINN model, which utilizes three types of loss functions: AQI penalty, data loss, and physics loss. Predictions from the PINN model are further classified using a structured mapping technique. Additionally, it is interpreted using XAI techniques, such as LIME, SHAP, and partial dependency plots. The model’s performance is evaluated using specific evaluation metrics, such as accuracy, precision, recall, and F1 score. Additionally, a comparative analysis is provided for conventional, ensemble machine learning (ML), and deep learning (DL) models.

### Physics-Informed neural networks for regression

PINN is a class of DL models that integrate physical laws into the training process [[24], [25], [26]]. These include differential equations or other known physical principles to guide the learning process, ensuring that the model learns in consideration of these laws and principles. This is particularly employed in regression problems, where the goal is to predict continuous values while maintaining consistency with physical laws. These can be used to model the relationship between various air quality parameters and AQI, accounting for the physical processes that govern air pollution.

The training of a PINN typically involves minimizing the following loss function in (1):

(1) $$L_{\text{total}}=L_{\text{data}}+L_{\text{physics}}$$

Where:

• $L_{\text{data}}$ is the data-driven loss.
• $L_{\text{physics}}$ is the physics-driven loss, which ensures that the model satisfies the governing physical equations.

The use of PINN enhances prediction accuracy while also improving model generalization and interpretability. In the case of AQI prediction, PINN ensures that the learned relationships between pollutants and AQI remain consistent with established environmental science, resulting in more accurate and reliable outcomes.

### AirSense-X approach

The primary objective of employing PINN is to enhance AQI prediction by integrating physical laws with data-driven learning. The approach used in this study utilizes both observed data and domain knowledge, helping the model to generalize more effectively. To achieve this, a novel total loss function as defined in (2):

(2) $$L=L_{\text{data}}+\;\lambda_{1}.L_{\text{aqi}}\;+\;\lambda_{2}.L_{\text{physics}}$$

Where $L_{\text{data}}\;$ensures that the model’s predictions align with real AQI values, computed using MSE is represented in (3):

(3) $$L_{\text{data}}=\frac{1}{N}\sum_{i=1}^{N}{\left(\text{AQ}I_{\text{predicted}}-\text{AQ}I_{\text{actual}}\right)}^{2}$$

Meanwhile, $L_{\text{aqi}}\;$ensures that the predicted AQI values follow the mathematical rules for AQI classification by applying a penalty when the predictions deviate from the rules.

(4) $$L_{\text{aqi}}=\sum_{i=1}^{N}{\left(\text{AQ}I_{i}-F\left(C_{i}\right)\right)}^{2}$$

In (4), where $L_{\text{aqi}}$ represents pollutant concentration and $F(C_{i})$ is the predefined AQI function. The function $F(C_{i})$ is determined using the official AQI calculation formula, which maps pollutant concentration C to its corresponding AQI. The PINN component ensures physics consistency by learning the governing equations, thereby preventing the model from making incorrect predictions.

For a given pollutant, the AQI is computed using the official formula as shown below:

(5) $$\text{AQ}I_{\text{pollutant}}=\frac{I_{\text{Hi}}-I_{\text{Lo}}}{B_{\text{Hi}}-B_{\text{Lo}}}.\left(C-B_{\text{Lo}}\right)+I_{\text{Lo}}$$

In (5),

• C: Observed Concentration
• $B_{\text{Hi}},\;B_{\text{Lo}}:\;$Breakpoint Concentration Range within which C lies.
• $I_{\text{Hi}},\;I_{\text{Lo}}:$ AQI values corresponding to the breakpoints.

In this study, physics loss is derived from the Advection-Diffusion-Reaction (ADR) equation, which models the temporal evolution of pollutant concentrations that influence the Air Quality Index (AQI). This Partial Differential Equation (PDE) captures how pollutants are transported by advection, spread through diffusion, and transformed or removed using chemical reactions. The ARD equation is simplified in this study due to the absence of wind or advection data in the dataset. The equation is designed to help the model to learn the relationship between pollutant concentrations and their changes over time. The PINN primarily approximates a continuous function that takes the time variable as input and produces the predicted AQI value based on the vector of pollutant concentrations, given the absence of time-stamped observations and spatial coordinates in the AQI dataset. To manage this, a time variable is introduced artificially as a sequential index, while the spatial diffusion term is assumed to be negligible. During training, the PINN simultaneously uses the observed AQI data and minimizes the residual. The physics loss function is defined in (6):

(6) $$L_{\text{physics}}=\sum \left(\frac{d\hat{C}}{\text{dt}}=D.\frac{d^{2}\hat{C}}{dx^{2}}+R\left(x\right)\right){\;}^{2}$$

Where:

• $\hat{C}$: The predicted pollutant concentration using PINN.
• $\frac{d\hat{C}}{\text{dt}}$: The rate of change of concentration over time.
• D:Diffusion coefficient (In this study, this is treated as constant due to the lack of spatial data).
• $\frac{d^{2}\hat{C}}{dx^{2}}$: Second-order spatial diffusion term (In this study, this term is neglected).
• R(x):Reaction term capturing changes in pollution levels due to environmental factors like chemical reactions.

As a result, the PINN focuses on learning the reaction-like behavior through pollutant interactions that influence AQI. The residual of the physics model incorporated into the loss function helps the neural network generalize better by leveraging domain knowledge to enhance accuracy and interpretability.

The physics-guided approach in PINN enhances AQI prediction accuracy by reducing the risk of overfitting. The integration of domain knowledge improves the model’s generalization capability to unseen scenarios, enhancing both predictive performance and the trustworthiness of the classification process.

On the other hand, a structure mapping technique is employed for classification tasks, where it processes air quality-related data instances to classify AQI into different categories, such as Good, Moderate, and others, based on the predicted AQI values by PINN. Overall, the AirSense-X approach for numerical AQI prediction and classification ensures that this approach achieves higher accuracy and enhanced interpretability in the presence of physical laws and environmental factors.

### Key hyperparameters for physics-informed neural networks

The performance and reliability of PINN significantly depend on the selection of hyperparameters. Manual grid search optimization was performed to fine-tune critical parameters such as learning rate, model architecture and training configuration.

Table 3 presents the hyperparameters to enhance the learning and generalization ability of the PINN model. The model architecture contains four hidden layers with a gradually decreasing number of neurons, which helps capture complex patterns while reducing the risk of overfitting. The LeakyReLU activation function is employed to allow a slight gradient for negative inputs, preventing the issue of dying neurons. To further regulate the model. A dropout rate of 0.2 is included along with L2 regulatization. Similarly, the loss function contains two subcomponents, AQI loss and physics-based loss, weighted at 0.7 and 0.3, respectively. This dual objective formulation ensures that the prediction error is reduced to a minimum with the help of physical laws. The training is optimized using the Adam optimizer with an initial learning rate of 0.001. The model is trained over 200 epochs.

**Table 3:** Key hyperparameters.

Category	Hyperparameter	Value
Model Architecture	Number of Hidden Layers	4
	Neurons per Layer	[256, 128, 64, 32]
	Activation Function	LeakyReLU(α=1)
	Dropout Rate	0.2
	Regularization	L2 regularization (λ=0.001)
Loss Function	$\lambda_{1}\;$(AQI loss)	0.7
	$\lambda_{2}\;$(Physics loss)	0.3
Optimizer and Learning	Optimizer	Adam
	Initial Learning Rate	0.001
Training Configuration	Epochs	200

### Explainable AI for interpretability

XAI techniques enhance the transparency and interpretability of complex models, such as PINN—CNN, which consider physical laws. These techniques help to identify which factors influence the predicted AQI, improving trust and decision-making [[27], [28], [29]].

### Local interpretable model-agnostic explanations (LIME)

LIME is a method used to interpret black-box models by approximating them with a simpler, interpretable model locally around a prediction [30]. It generates perturbed samples near the instance of interest and trains a surrogate model to approximate the original model’s decision. The objective function is defined as:

(7) $$L\left(f,\;g,\;\pi_{x}\right)=\sum_{z\in Z}\pi_{x}\left(z\right).{\left(f\left(z\right)-g\left(z\right)\right)}^{2}+\Omega \left(g\right)$$

In (7), fis the original black box, gis the interpretable surrogate model, and $\pi_{x}\left(z\right)$ is a measure between the perturbed instance and the instance of interest. The loss function L measures how well it approximates in the local neighborhood of x, while the regularization term Ω(g) ensures that gremains interpretable and straightforward.

In this study, LIME is used to interpret the AQI predictions. PINN remains a black-box model despite its ability to incorporate physical laws and learn interactions between pollutants. LIME is employed to identify pollutants that significantly influence AQI prediction. This interpretability is crucial in the environmental context, as it provides transparency and enables everyone to understand the rationale behind each prediction, fostering better policy-making and public communication.

### Shapley additive exPlanations (SHAP)

SHAP uses game theory to assign a value to each feature, reflecting its contribution to a model’s prediction [31,32]. It computes Shapley values, which are the weighted average of the marginal contributions of each feature over all possible subsets of features as defined in (8):

(8) $$\begin{array}{c} \phi_{i}\left(f\right)=\sum_{S\leq N\left\{i\right\}}\frac{\left|S\right|!\left(\left|N\right|-\left|S\right|-1\right)!}{\left|N\right|!}[f\left(S\cup \left\{i\right\}-f\left(S\right)\right)] \end{array}$$

Here, $\emptyset_{i}\left(f\right)$ is the Shapley value for the feature, and f(s) represents the model’s prediction with the feature in subset S.

In this study, SHAP is utilized to interpret the predictions of the PINN model for AQI prediction. While PINN utilizes physical equations for more reliable modeling, SHAP offers both global and local explanations for feature importance. This level of interpretability enables domain experts and policymakers to identify the most significant pollutants affecting air quality.

## Algorithms

### Algorithm for PINN model

Algorithm 1 *illustrates the implementation of the PINN approach for AQI prediction, which integrates physical laws with data-driven learning to enhance both accuracy and interpretability. This approach begins by taking input on pollutant concentration and actual AQI values, which are then split into training and testing sets. The PINN model is initialized as a neural network function that learns not only from the data but also from physical laws governing AQI. A novel total loss function is developed, consisting of three components: data loss, AQI penalty loss, and physics-based loss, each weighted by hyperparameters. The data loss ensures that predictions are aligned with the absolute AQI values using the Mean Squared Error (MSE). The AQI penalty loss ensures that predicted values are calculated using AQI calculation formulas by penalizing the derivations from pollutant-to-AQI mappings. Physics loss is derived from a simplified ADR equation, which captures how pollutants evolve over time. Due to the lack of spatial data, the diffusion term is considered negligible, and the PINN focuses on learning the temporal and reaction-like dynamics of the pollutant. The model is trained to minimize the total loss using an optimizer, effectively learning both trends and domain rules. Once trained, the model can predict AQI values that are not only accurate but also physically meaningful and relevant to the context. This approach significantly improves generalization ability, reducing the risk of overfitting and enhancing the performance of predictions, making it a strong solution for environmental monitoring and decision-making.*

**Algorithm 1:** Algorithm for PINN Model.

*Input:*$D={\left\{\left(X_{i},\;y_{i}\right)\right\}}_{i=1}^{N}$*(AQI Dataset)*
*Output: Predicted AQI value*
*1. Define Total Loss function:*
*2. Define Sub-Loss functions:* *2.1. Data Loss (Mean Squared Error)* $L_{data}=\frac{1}{N}\sum_{i=1}^{N}{\left(AQI_{predicted}-AQI_{actual}\right)}^{2}$ *2.2. AQI Penalty Loss* $L_{aqi}=\sum_{i=1}^{N}{\left(AQI_{i}-F\left(C_{i}\right)\right)}^{2}\;$ *Where*$F(C_{i})$*is the official AQI function as shown:* $AQI_{pollutant}=\frac{I_{Hi}-I_{Lo}}{B_{Hi}-B_{Lo}}.\left(C-B_{Lo}\right)+I_{Lo}$ *2.3. Physics Loss* *Let*$\hat{C}\;$*be the predicted pollutant concentration*
*3. Initialize parameters:* *4. Set* $\theta_{o}$ *randomly for the PINN*
*5. For each iteration t = 0 to T – 1 do* *5.1. Computer total loss:* $L_{PINN}=L_{data}+\;\lambda_{1}.L_{aqi}\;+\;\lambda_{2}.L_{physics}$ *5.2. Computer gradient:* $\nabla_{\theta }L_{PINN}$ *5.3. Update parameters:* $\theta_{t+1}=\theta_{t}-\eta .\nabla_{\theta }L_{PINN}$ *End for*
*6. Set final model parameters:* $\theta_{final}=\theta_{t}$
*7. Return* $\theta_{final}$

### Algorithm for explainable AI

Algorithm 2 *applies XAI techniques to interpret model predictions. It first uses LIME to approximate the model locally by minimizing error with regularization and then applies SHAP to calculate feature importance by comparing predictions with and without each feature. The algorithm takes the trained model and test data as input and generates explanations using two interpretability methods, LIME and SHAP. LIME approximates the complex model locally by generating perturbations around the input data and training a simpler interpretable model to minimize the combined prediction error and regularization. This helps identify the input features that have a significant influence on the model’s predictions. Similarly, SHAP assigns Shapley value to each feature, based on cooperative game theory, which provides a feature’s contribution by comparing the model’s output with and without the feature for all possible combinations. These two techniques, when combined, offer both local and global explanations, thereby enhancing the transparency, trust, and accountability of the AQI prediction model.*

**Algorithm 2:** Algorithm for Explainable AI.

*Input: Trained model, test data*$D_{test}=\left\{x_{1},\;x_{2},\;x_{3}\ldots .\;x_{n}\right)\;$
*Output: Interpretability Explanations*ε
1. Apply LIME 2. Generate perturbed samples $Z={\left\{z_{j}\right\}}_{j=1}^{m}\;$around $x_{i}$ 3. Obtain corresponding predictions $f(z_{j})$ 4. Define local surrogate model g(z)≈f(z), typically linear 5. Solve optimization: $L_{LIME}=\arg min\sum_{z\in Z}\pi_{x}\left(z\right).{\left(f(z)-g(z)\right)}^{2}+\Omega \left(g\right)$ 6. Extract explanation
*7. Apply SHAP* *a. For each feature* i={1,2.…d},computemarginal *b. Aggregate SHAP values into explanation:* $\varepsilon_{SHAP}\left(x_{i}\right)=\left\{\phi_{1},\phi_{2}\ldots \;\phi_{d}\right)\;$
*8. Store final explanations* $\varepsilon \left(x_{1}\right)=\left\{\varepsilon_{LIME}\left(x_{i}\right),\;\varepsilon_{SHAP}\left(x_{i}\right)\right\}$
*9. End for*
*10. Return Instance level explanations*

### Method validation

*The proposed AirSense-X approach is evaluated using evaluation metrics such as accuracy, precision, recall, and F1 Score. The PINN model for regression is evaluated using several evaluation metrics, including Root Mean Square Error (RMSE), Mean Square Error (MSE), and Mean Absolute Error (MAE). Additionally, the performance of the proposed approach is compared with that of conventional, ensemble machine learning (ML), and deep learning (DL) models.*

Table 4 *compares the performance of different regression models based on RMSE, MSE, and MAE. Linear Regression showed the highest error values, indicating poor predictive performance, while Support Vector Regressor and Random Forest Regressor significantly reduced errors, showing better performance. PINN outperforms all other models, achieving the lowest MSE of 2.86, MAE of 1.10, and RMSE of 1.69, which highlights its superior performance by integrating data-driven learning with physical constraints.*

**Table 4:** Comparative analysis with regression models.

*Model*	*MSE*	*RMSE*	*MAE*
*Linear Regression*	*2831.43*	*53.21*	*33.85*
*Support Vector Regressor*	*210.32*	*14.50*	*9.85*
*Random Forest Regressor*	*112.67*	*10.61*	*7.92*
*PINN*	*2.86*	*1.69*	*1.10*

**Fig. 3:**
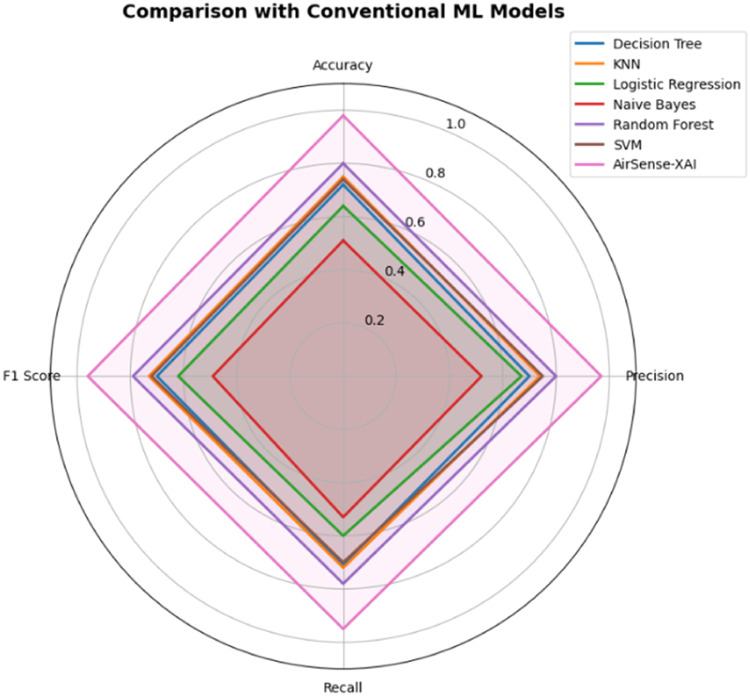
Comparison with Conventional ML Models.

Table 5 *and* Fig. 3 *compare AQI classification models with Random Forest, achieving accuracies of 80 %, 75 % by KNN, and 74 % by SVM. Decision Tree shows moderate performance with 72 % accuracy, while Logistic Regression achieves an accuracy of 64 %. Among all the models, Naive Bayes achieved the least accuracy, with 51 %. The proposed AirSense-X model significantly outperforms all other models, achieving an accuracy of 98 %, which highlights its superior performance and reliability in AQI classification.*

**Table 5:** Comparative analysis with conventional ML models.

*Model*	*Accuracy*	*Precision*	*Recall*	*F1 Score*
*Decision Tree*	*0.72*	*0.70*	*0.71*	*0.70*
*KNN*	*0.75*	*0.74*	*0.72*	*0.73*
*Logistic Regression*	*0.64*	*0.67*	*0.60*	*0.62*
*Naive Bayes*	*0.51*	*0.52*	*0.53*	*0.49*
*Random Forest*	*0.80*	*0.80*	*0.78*	*0.79*
*SVM*	*0.74*	*0.75*	*0.70*	*0.72*
*AirSense-X*	*0.98*	*0.97*	*0.95*	*0.96*

*The confusion matrices of several conventional machine learning models are illustrated in* Fig. 4*. It is observed that the Decision Tree and KNN models exhibited moderate performance, with misclassification rates of 4832 and 5104, respectively. Logistic Regression and Naive Bayes performed poorly, with misclassification rates of 7072 and 8255 instances, respectively. SVM performed moderately with 6800 misclassifications, while Random Forest performed the best with 15,356 correct classifications.*

**Fig.. 4:**
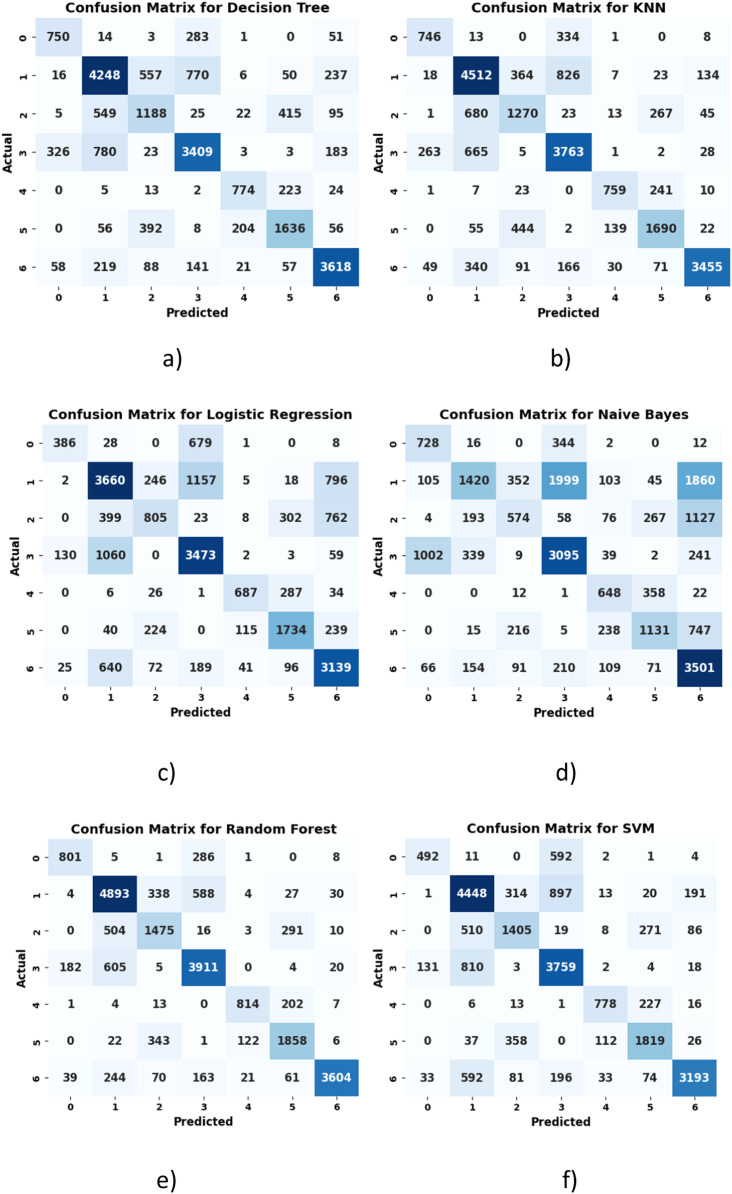
Confusion Matrix of a) Decision Tree b) KNN c) Logistic Regression d) Naive Bayes e) Random Forest f) SVM.

**Fig. 5:**
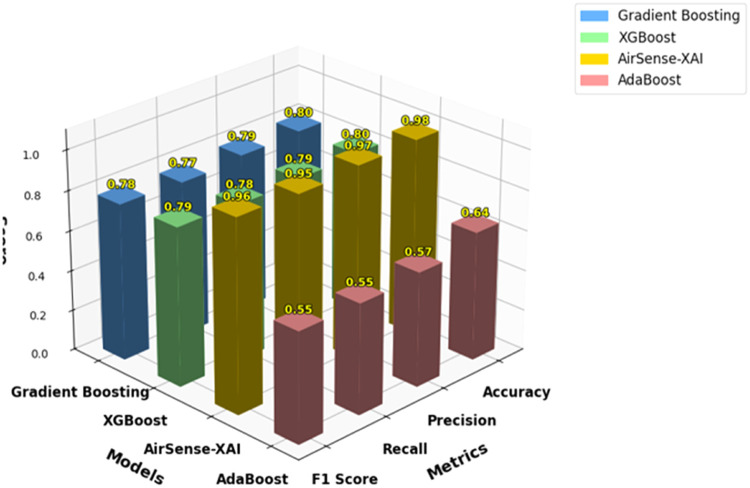
Comparison with ensemble ML models.

Table 6 *and* Fig. 5 *compare the performance of AdaBoost, Gradient Boosting, XGBoost, and AirSense-X. Gradient Boosting and XGBoost are observed to show better performance, achieving 80 % accuracy and strong precision, with recall and F1 scores of around 78 %. AirSense-X outperforms all other models, achieving 98 % accuracy, a precision of approximately 97 %, a recall of 95 %, and an F1 score of 96 %, demonstrating superior overall performance.*

**Table 6:** Comparative analysis with ensemble ML models.

*Model*	*Accuracy*	*Precision*	*Recall*	*F1 Score*
*AdaBoost*	*0.64*	*0.57*	*0.55*	*0.55*
*Gradient Boosting*	*0.80*	*0.79*	*0.77*	*0.78*
*XGBoost*	*0.80*	*0.79*	*0.78*	*0.79*
*AirSense-X*	*0.98*	*0.97*	*0.95*	*0.96*

**Fig. 6:**
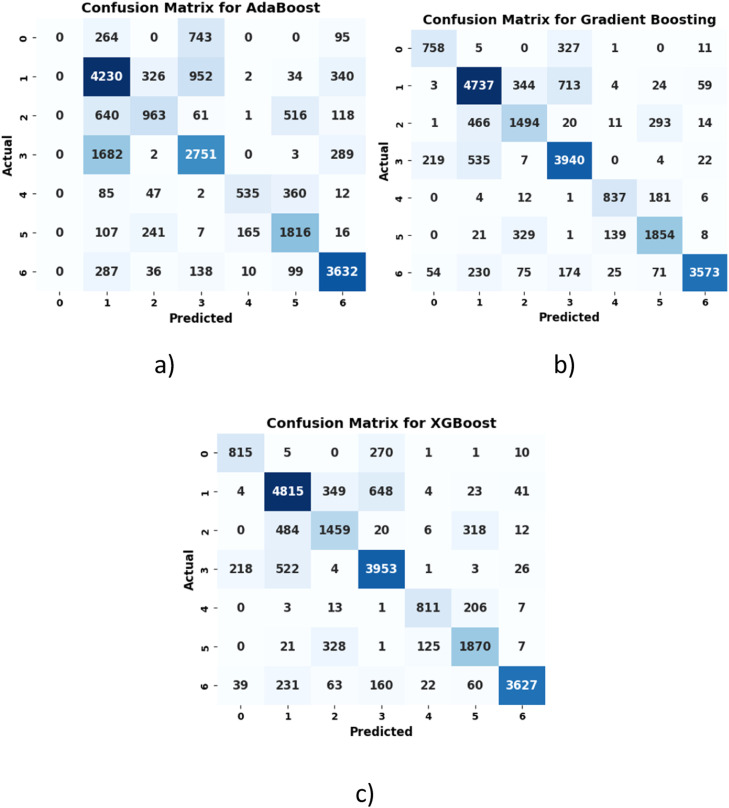
Confusion matrix of a) AdaBoost b) gradient boosting c) XGBoost.

Fig. 6 *illustrates the confusion matrix of AdaBoost, Gradient Boosting, and XGBoost. It is observed that the AdaBoost model correctly classified 14,172 instances but struggled with class imbalance, particularly for class 0. The Gradient Boosting model is observed to perform better by correctly classifying 16,952 instances with fewer misclassifications. Similarly, the XGBoost model outperformed other ML models by correctly classifying 17,082 instances with only 2918 misclassifications.*

**Fig. 7:**
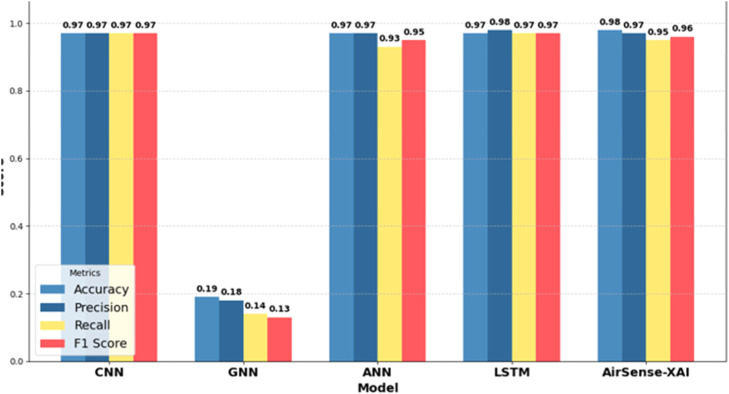
Comparison with DL models.

*The comparative analysis of DL models, based on evaluation metrics, is presented in* Table 7 *and* Fig. 7*. CNN, ANN, and LSTM each achieve an accuracy of 97 %, with LSTM achieving the highest precision of 98 %. In contrast, GNN performs poorly with 19 % accuracy, likely due to its unsuitability for this task. The proposed AirSense-X model outperforms all other models with 98 % accuracy, demonstrating its superior performance among all deep learning models.*

**Table 7:** Comparative analysis with DL models.

*Model*	*Accuracy*	*Precision*	*Recall*	*F1 Score*
*CNN*	*0.97*	*0.97*	*0.97*	*0.97*
*GNN*	*0.19*	*0.18*	*0.14*	*0.13*
*ANN*	*0.97*	*0.97*	*0.93*	*0.95*
*LSTM*	*0.97*	*0.98*	*0.97*	*0.97*
*AirSense-X*	*0.98*	*0.97*	*0.95*	*0.96*

**Fig. 8:**
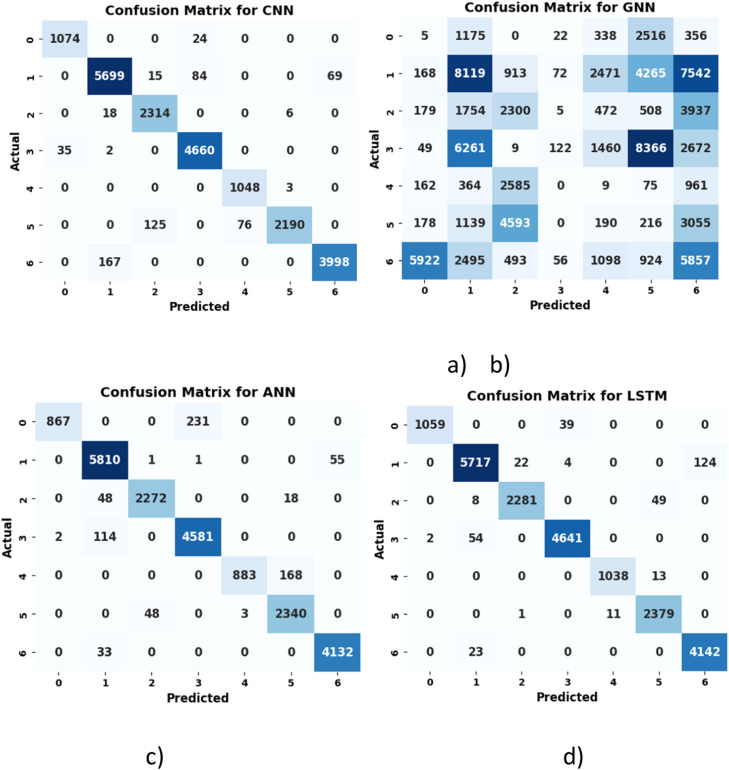
Confusion matrix of a) CNN b) GNN c) ANN d) LSTM.

Fig. 8 *illustrates that the CNN model correctly classified 19,117 instances, with 612 misclassifications, primarily in classes 1, 2, and 6. The ANN model achieved 19,615 correct classifications but misclassified 639 instances while struggling with classes 0 and 4. Similarly, the LSTM model correctly classified 19,257 instances, with 616 misclassified instances, particularly in classes 0, 1, and 2. Additionally, it is observed that the GNN model had 27,004 incorrect classifications, especially in classes 1, 2, and 5, which highlights its poor performance.*

**Fig. 9:**
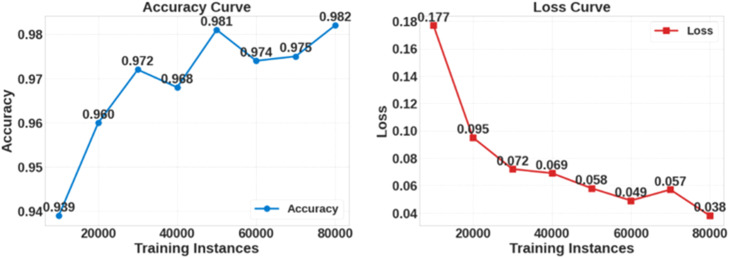
Accuracy and loss curve for PINN model.

**Fig. 10:**
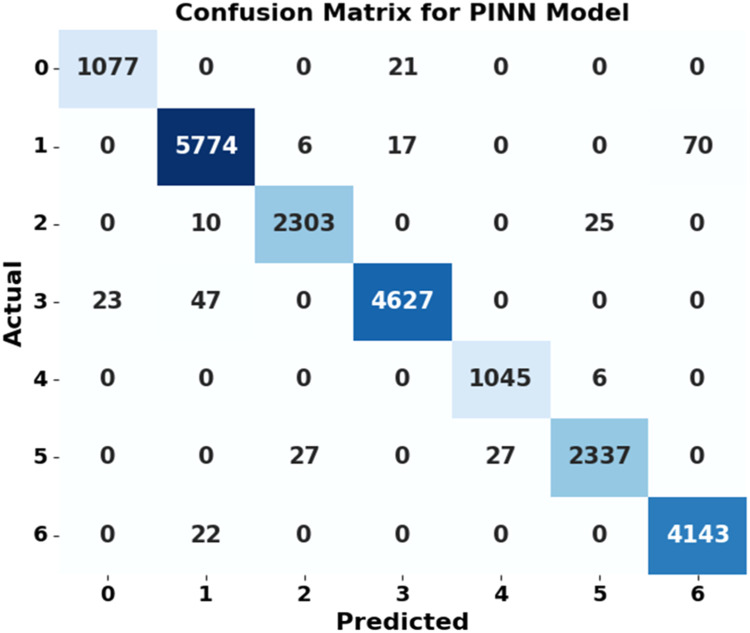
Confusion matrix of PINN model.

*The performance of the proposed PINN model is illustrated in* Fig. 9*. The accuracy graph in the figure shows that the accuracy improves from 0.94 to 0.98 as the number of training instances increases from 10,000 to 80,000.* Fig. 10 *shows the confusion matrix for the PINN model, which resulted in 21,306 correct predictions and 268 incorrect predictions.*

Table 8 *provides a comparative analysis of the performance of the PINN model using different loss function configurations. The data loss results initially show the fastest training, but a relatively lower MSE and R2 score. Adding physics loss introduces significant physical constraints but reduces accuracy and training time. Additionally, including AQI loss enhances accuracy but with slightly longer training time. Finally, the hybrid loss, which combines the data loss, AQI loss and physics loss, achieves the best overall performance with the lowest MSE and R2 scores, showing superior performance.*

**Table 8:** Ablation study: altering the loss functions.

*Loss Functions*	*MSE*	*R^2^ score*	*Training Time (mins)*	*Finding*
*Only Data Loss*	*0.0191*	*0.9329*	*0.75*	*Fast Training*
*Data Loss + Physics Loss*	*0.0249*	*0.8927*	*4.11*	*Added constraints, reduced accuracy*
*Data Loss + AQI Loss*	*0.0189*	*0.9231*	*4.88*	*Slower training and slight accuracy gain*
*Hybrid Loss (Data Loss + AQI Loss + Physics Loss)*	*0.0084*	*0.9528*	*5.65*	*Longer training and best performance*

Table 9 *presents a comparative analysis of the PINN model on different cities' numerical AQI prediction datasets. Among all the towns, Dubai achieved the lowest errors and the highest R2 score, indicating the best performance. London showed the highest errors along with a slightly lower R2 score. The model performs well across all cities, maintaining strong accuracy and reliability.*

**Table 9:** Ablation study: altering the datasets.

*City*	*MSE*	*RMSE*	*R^2^ score*
*India*	*0.0084*	*0.0916*	*0.9528*
*Dubai*	*0.0072*	*0.0849*	*0.9671*
*London*	*0.0105*	*0.1025*	*0.9341*
*New York*	*0.0091*	*0.0954*	*0.9422*

**Fig. 11:**
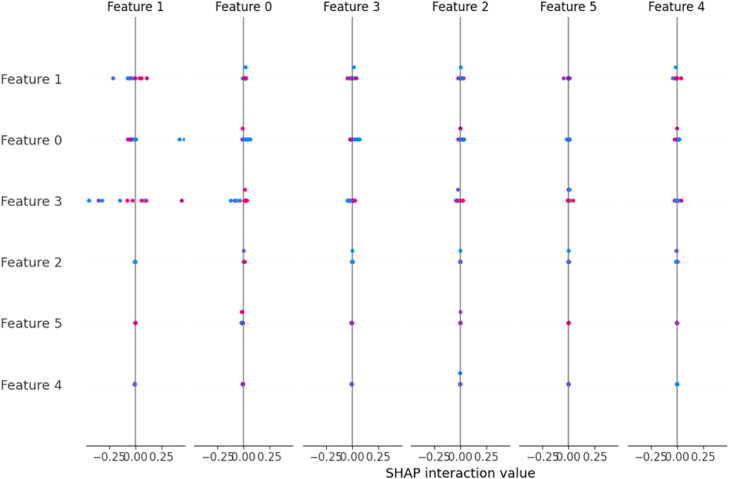
SHAP interaction plot.

*The plots obtained using XAI techniques are illustrated in* Figs. 11*,* 12*, and* 13*. The SHAP interaction plot in the figure highlights the key feature interactions affecting the predictions, showing how different features contribute to classification outcomes. Similarly, the bar chart in the figure shows that the features PM10, AQI, NH3, and NOx have a negative impact on classification. In contrast, PM2.5 has a positive influence on predicting the “Moderate” AQI class. Additionally, the figure illustrates the partial dependence plots, which reveal that the model predictions vary significantly with changes in feature values, indicating their strong influence on classification.*

**Fig. 12:**
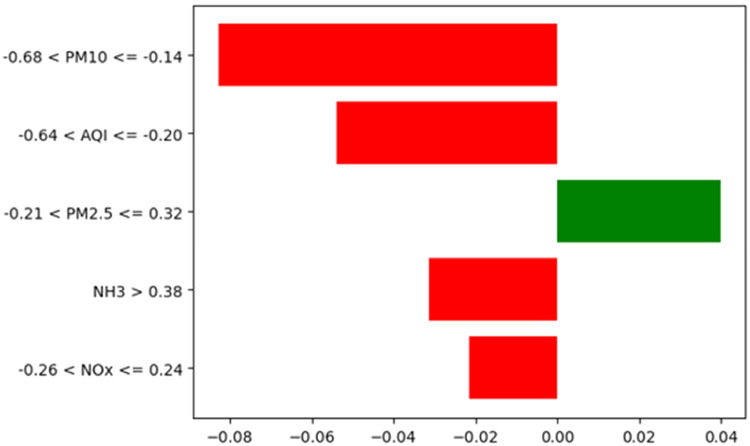
Local explanations chart.

**Fig. 13:**
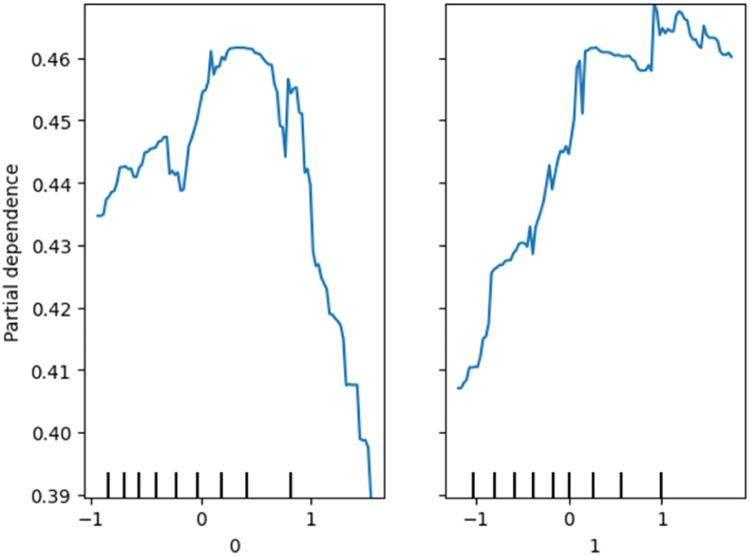
Partial dependence plots.

*The results indicate that PINN achieves the lowest MSE of 2.86, RMSE of 1.69, and MAE of 1.10, highlighting its ability to integrate data-driven learning with physical constraints for enhanced prediction accuracy. Additionally, the accuracy and loss curves for the PINN—CNN hybrid model show a stable improvement in accuracy, from 94 % to 98 %, as the number of training instances increases from 10,000 to 80,000. The confusion matrix for this hybrid model indicates that the model correctly classifies 21,306 instances with only 268 misclassifications, highlighting its reliability and accuracy. These findings demonstrate that the PINN and the hybrid model effectively minimize errors and improve classification accuracy compared to conventional, ensemble, and deep learning (DL) models.*

*Air pollution remains a critical global challenge, posing severe health and environmental concerns. The increasing concentration of pollutants such as PM10, PM2.5* *m Nox, and NH3 significantly impacts air quality, leading to respiratory diseases, climate change, and reduced lifespan. Traditional air quality monitoring systems often lack predictive accuracy and fail to provide real-time insights, requiring a more reliable and interpretable solution. To address these limitations, this study proposed AirSense-X, an approach that employs PINN along with XAI for enhanced interpretability. The study employs PINN for regression, utilizing XAI techniques such as SHAP and LIME for interpretation. The results indicate that the PINN model outperforms other models in regression, achieving the lowest error values, including an MSE of 2.86, an RMSE of 1.69, and an MAE of 1.10. Similarly, conventional and ensemble ML models were evaluated using the proposed approach, with AirSense-X outperforming all the models with 98 % accuracy.*

*Furthermore, a comparative analysis with deep learning (DL) models highlighted the proposed approach’s superior performance in terms of accuracy, achieving 98 % precision, 95 % recall, and a 96 % F1 score. This study benefits government bodies, urban planners, industrial pollution control boards, and health organizations by providing an advanced, interpretable AI-driven air quality monitoring system. Future works could involve focusing on real-time data integration for dynamic AQI predictions. Additionally, incorporating IoT-based sensors would help with real-time AQI predictions, making AirSense-X a stronger and more reliable solution for global air quality management.*

## Conclusion

*Air pollution remains a pressing global issue, significantly affecting human health, climatic conditions and natural ecosystems. Accurate prediction of AQI is necessary for enabling the proactive environmental management public health interventions. However, traditional prediction models face challenges in modeling the dynamic and non-linear behavior of air pollution which is governed by complex physical phenomena. To address these challenges, there is a growing need for intelligent systems that include the use of domain-specific knowledge such as the physical loaws goverming pollutant disperson and transformation along with learning from the data. This highlights the necessity for a solution that bridges data-driven learning, domain-specific knowledge and physical reasoning. This study introduced AirSense-X approach which combines PINN with XAI techniques to enhance AQI prediction and ensure reliability. The experimental results validate the effectiveness of the proposed approach. The results indicate that the AirSense-X approach achieved an accuracy of 98 % with precision of 0.97, recall of 0.95 and F1-score of 0.96. The proposed approach ensures better interpretability and transparency for enhancing trust in the model’s decision-making process. This study could benefit environmental scientists, public health officials and policy makers for better air quality management and regulatory planning.*

## Limitations and future works

*Despite the promising performance of the proposed AirSense-X approach, the study has few limitations. Firstly, the model relies on a static historical dataset sourced from fixed monitoring stations, which may not accurately reflect fluctuations in pollution levels or sudden spikes caused by localized events or changes in weather conditions. This limitation restricts the model’s ability to provide dynamic, real-time predictions. Additionally, the dataset is limited to specific regions within India, limiting its generalization ability. Future work for this study would involve addressing these challenges and integrating real-time data through IoT-based air quality sensors, enabling more accurate AQI predictions. Furthermore, the model will be expanded to include additional environmental factors, such as temperature, humidity, and wind speed, which are crucial for capturing the nature of air pollution and enhancing the model’s performance in various real-world scenarios.*

## Related research article

*None.*

## For a published article

*None.*

## Ethics statements

*Informed consent was obtained from all participants. The study followed institutional ethical guidelines and ensured participant anonymity and data confidentiality.*

## CRediT author statement

Sai Varun Chandrashekar – Methodology, Conceptualization, Writing – Original Draft. Firoz Khan – Data Curation, Validation, Visualization. Sunaina Sridhar and Rajesh Kumar Dhanaraj- Literature Survey, Visualization, Supervision. Dragan Pamucar: Project administration, Funding acquisition. Balamurugan Balusamy and Malathy Sathyamoorthy – Writing – Review & Writing, Visualization, Methodology

## Code availability

The code used in this study can be accessed using the following Google Colab link: https://colab.research.google.com/drive/151TRaTrLTKpIfg9Kgew4LcqKxkRtR1fj?usp=sharing

## Declaration of competing interest

The authors declare that they have no known competing financial interests or personal relationships that could have appeared to influence the work reported in this paper.

## Data Availability

Data will be made available on request.
